# Attributes of response in depressed patients switched to treatment with duloxetine

**DOI:** 10.1111/j.1742-1241.2010.02546.x

**Published:** 2011-01

**Authors:** D Sagman, D McIntosh, M S Lee, H Li, S Ruschel, N Hussain, R E Granger, A C Lee, J Raskin

**Affiliations:** 1 Lilly Research Laboratories, Eli Lilly CanadaToronto, ON, Canada; 2 Department of Psychiatry, University of British ColumbiaVancouver, BC, Canada; 3 Department of Psychiatry, Korea University College of MedicineSeoul, Korea; 4 Department of Psychiatry, Second Affiliated Hospital, Zhejiang University School of MedicineHangzhou, China; 5 Hospital Mário KröeffPsiquiatria, Brazil; 6 Intercontinental Information Sciences, Eli Lilly Australia Pty LtdMacquarie Park, NSW, Australia

## Abstract

**Background:**

This study was designed to assess clinical and functional outcomes associated with switching to duloxetine treatment in patients with major depressive disorder (MDD) experiencing emotional and painful physical symptoms in their current episode.

**Methods:**

In this 8-week, multinational, multicentre, single-arm, open-label clinical trial, 242 MDD patients were switched to duloxetine 60 mg/day after selective serotonin reuptake inhibitor (SSRI) or serotonin and norepinephrine reuptake inhibitor (SNRI) treatment. The primary analysis compared mean change from baseline in Brief Pain Inventory – Modified Short Form (BPI-SF) interference score between initial responders [≥ 50% reduction from baseline on the 17-item Hamilton Depression Rating Scale (HAMD_17_) Maier subscale] and initial non-responders after 4 weeks. Initial responders continued with duloxetine 60 mg/day. Initial non-responders received duloxetine 120 mg/day for the remaining 4 weeks. Depression, pain, anxiety and functional outcomes were also compared after 8 weeks.

**Results:**

BPI-SF interference decreased from baseline in initial responders (*n* = 108) and initial non-responders (*n* = 85) after 4 weeks of duloxetine treatment, with greater reductions in initial responders [BPI-SF mean difference in reduction: 1.01 (95% CI 0.42–1.61); p < 0.001]. Reductions in pain interference favouring initial responders were also apparent after 8 weeks [0.68 (95% CI: 0.03–1.33); p = 0.042]. Depression, pain, anxiety and function improved over 8 weeks across patient groups.

**Conclusions:**

Elements of core mood and pain are important residual symptoms following poor treatment response in MDD. Early improvement in these symptoms after switching to duloxetine indicated an increased chance of functional recovery.

**What’s known**

- Studies have shown that the presence of painful physical symptoms reduces the likelihood of remission in depressed patients.
- A relationship between the effective treatment of painful physical symptoms and depression remission rates has been recently shown.
- Duloxetine has demonstrated clinical improvements in painful physical symptoms associated with major depressive disorder.

**What’s new**

- This study explores the clinical course and functional outcomes of depressed patients, experiencing emotional and painful physical symptoms, who are switched to duloxetine treatment.
- The results highlight the importance of improvements in mood, pain, anxiety and functioning in the overall remission of patients with major depressive disorder.
- An early response in these symptoms after switching to duloxetine may improve the chances of a clinically meaningful, functional recovery.

## Introduction

Major depressive disorder (MDD) is a chronic, disabling condition encompassing emotional, behavioural and physical symptoms that impact considerably upon patients (1,2). Treatment for MDD aims to achieve complete remission of depressive symptoms and facilitate a return to normal functioning. Antidepressant medications, particularly selective serotonin reuptake inhibitors (SSRIs) and selective serotonin and norepinephrine reuptake inhibitors (SNRIs), are widely used as first-line treatment options for MDD. However, suboptimal response to antidepressant medication is common; up to 35% of patients treated in routine clinical practice have an inadequate response to first-line therapy (3) and only a third of patients may achieve clinical remission criteria (4,5).

Failure to achieve full remission of MDD is associated with a high risk of chronic symptoms and impaired quality of life (6–8) and physicians routinely switch antidepressant medications to improve clinical response (9). Although switching antidepressant medications is widespread in clinical practice, systematic evaluations of the consequent efficacy and tolerability outcomes are limited. Identifying key response attributes that enhance earlier recognition of patients who benefit from switching antidepressants would be of value.

Duloxetine hydrochloride (duloxetine) is a relatively balanced dual reuptake inhibitor of serotonin (5-HT) and norepinephrine (NE) (10,11). In a previous study, a switch to duloxetine (60–120 mg/day) following SSRI treatment failure produced significant improvements in emotional and physical symptoms of depression, irrespective of whether patients were switched to duloxetine abruptly or tapered off their prior SSRI whilst receiving concomitant duloxetine (9). In addition, duloxetine has demonstrated clinical improvements in painful physical symptoms (PPS) associated with MDD (12). Significantly greater reductions in PPS were shown with duloxetine vs. placebo after 8 weeks of treatment in MDD patients with at least moderate pain associated with their major depressive episode (13) and an independent analgesic effect in MDD has been proposed (14).

The aim of this current study was to focus on the attributes of response in MDD patients with at least moderate pain, further expanding on the available data on switching to duloxetine following partial or non-response to SSRIs.

The primary objective of this study was to investigate the change in pain interference [as represented by the Brief Pain Inventory – Modified Short Form (BPI-SF) interference score], relative to a change in core mood symptoms [as represented by the Maier subscale of the 17-item Hamilton Depression Rating Scale (HAMD_17_)]. The core emotional symptoms of depression represented by the HAMD_17_ Maier subscale include depressed mood, feelings of guilt, loss of interest in work and daily activities, psychomotor retardation, agitation and psychic anxiety (15). With the relatively balanced dual mechanism of action of duloxetine (16), it was hypothesised that patients who demonstrated improvements on the Maier subscale initially would show a higher degree of improvement in pain interference than patients who did not (1).

## Methods

### Study design

This multicentre, single-arm, open-label trial evaluated duloxetine in outpatients with MDD who failed to respond to one course of treatment with either an SSRI or SNRI antidepressant for the current depressive episode (at study entry).

Patients who met the study eligibility criteria were treated with open-label duloxetine, 60 mg/day, for 4 weeks (acute therapy period), after which they entered a further 4-week treatment period. Patients who did not initially respond on the Maier subscale during the acute therapy period received duloxetine 120 mg/day (dose-optimisation period) for an additional 4 weeks, whereas responding patients maintained the 60 mg/day dose. In accordance with the principles of the Declaration of Helsinki, all patients provided informed consent prior to the administration of any study drug.

Based on a previous duloxetine study, the proportion of patients demonstrating a Maier response when switched from an SSRI or SNRI to duloxetine for 4 weeks was estimated to be 44% and 22%, respectively (9). The estimated ratio of SSRI/SNRI patients entering this study was 60 : 40, with the overall response estimated at 35%.

Using data from another duloxetine study (17), the mean (SD) difference in BPI-SF interference score between initial responders and initial non-responders in the acute therapy phase was estimated to be −1.64 (2.8). Approximately 240 patients with a 25% dropout rate was calculated to provide 90% power at a response rate of 25% and provide 96% power for a response rate of 35%.

### Selection of patients

Study participants were outpatients from 22 sites in Brazil, Canada, China and Korea, aged 18 years or older, who met the Diagnostic and Statistical Manual for Mental Disorders, 4th Edition, Text Revision (DSM-IV-TR) (18) disease diagnostic criteria for MDD. Patients were receiving either an SSRI or SNRI antidepressant prescribed for depression treatment at locally recommended doses, for at least 4 weeks prior to study entry. A HAMD_17_ score ≥ 15 (19), Clinical Global Impression of Severity (CGI-S) score ≥ 3 and BPI-SF interference score ≥ 3 were required at screening and baseline.

Patients were ineligible to participate if they met any of the following criteria: a current primary Axis I disorder other than MDD; a history of substance abuse or dependence; any organic pain syndrome or continuous analgesic use for chronic pain; pregnancy or breastfeeding; and previous failure with duloxetine treatment or treatment-resistant depression (20). Patients who were at suicidal risk, or had a serious medical condition likely to require hospitalisation and/or use of excluded medications were also excluded.

### Treatments administered

Patients received duloxetine 60 mg/day, administered orally with food following a direct switch from an SSRI or SNRI antidepressant (with the exception of fluoxetine, which had to be discontinued for a minimum of 28 days prior to baseline). After 4 weeks, patients who responded to duloxetine (≥ 50% reduction from baseline on the Maier subscale of the HAMD_17_; ‘initial responders, IR’) continued to receive 60 mg/day for the remaining 4 weeks. Patients who did not respond to duloxetine in this initial 4-week period (< 50% reduction from baseline on the Maier subscale of the HAMD_17_; ‘initial non-responders, INR’) received duloxetine 120 mg/day for the remainder of the study.

All concomitant medications taken during the study were recorded. Patients were excluded from taking any antidepressant other than duloxetine. Patients requiring continuous use of analgesics (>Step 2 of the WHO definition) because of chronic pain for greater than 6 months were excluded from the study. Episodic use of some analgesics, non-steroidal anti-inflammatory drugs (NSAIDs) and narcotics was allowed if used to treat acute injury or surgical procedure for no longer than six consecutive days.

### Clinical and functional outcomes and safety evaluations

The primary objective of this study was to compare the mean change in BPI-SF interference score from baseline to week 4 between the IR and INR groups. A secondary focus was the BPI-SF interference score in responders and non-responders at week 8. The terms ‘responders’ and ‘non-responders’ were used to describe the 8-week secondary outcomes of the IR and INR groups.

Other secondary measures were: longitudinal assessments of mean baseline to week 8 change in HAMD_17_ total score and Maier subscales; the Hamilton Anxiety Rating Scale (HAM-A) total score and subscales; the BPI-SF average pain score; the CGI-S score; the Patient Global Impression of Improvement (PGI-I) score; and the Sheehan Disability Scale (SDS).

The proportion of patients achieving HAMD_17_ Maier response (≥ 50% reduction from baseline); HAMD_17_ Maier onset (≥ 20% improvement from baseline); HAMD_17_ Maier sustained response (Maier response sustained through the end of the study); HAMD_17_ total response (≥ 50% reduction from baseline) and sustained response (total response sustained through the end of the study); HAMD_17_ total remission (total score ≤ 7) and sustained remission (total score ≤ 7 sustained through the end of the study); BPI-SF interference onset score (≥ 30% improvement); BPI-SF interference score with ≥ 50% improvement, at 4 weeks and 8 weeks; and the time to onset of these criteria were also assessed.

Dose-optimisation in the INR group was assessed using HAMD_17_ Maier subscore and HAMD_17_ total score response rates, as well as the HAMD_17_ remission rate.

All adverse events were reported during the study period using spontaneously reported treatment-emergent adverse events (TEAEs), discontinuations due to adverse events (AEs) and vital signs. Safety measures included physical examination, blood pressure and heart rate, pre-existing conditions and concomitant medications. Laboratory measurements included haematology, chemistry and electrolytes, urinalysis, urine pregnancy test and urine drug screen.

The measures used in this study have been documented and validated in the literature and are generally regarded as reliable and relevant tools for the assessment of MDD patients. All study investigators were trained in the proper administration of each scale.

### Statistical analyses

Data of all enrolled patients were included in the statistical analyses. All patients who received at least one dose of study drug were included in the safety analyses. Patients with significant protocol violations or non-compliance were excluded from the outcomes analyses. Patients who received incorrect dose-optimisation at week 4 were included in outcomes analyses up to the time of dose-optimisation. Comparisons between initial responders and initial non-responders, as classified at week 4, were made for most of the outcomes analyses.

Two-sided significance levels of 0.05 and 0.10 were defined *a priori* to evaluate group- and interaction-effects, respectively. No formal adjustments were made for multiple comparisons. No imputations were conducted for missing covariates. Descriptive statistics were used to characterise patients at study entry. A two-sample *t*-test was used to compare continuous variables and Fisher’s exact test for categorical variables. Covariate adjustment, including prespecified known potential confounders as fixed, categorical effects of gender, group (IR, INR and responders, non-responders), country, visit number, previous therapy (SSRI/SNRI), reason for switch, response group-by-visit interaction, response group-by-country interaction, as well as continuous covariates of age and baseline score, were made to control for baseline imbalances for all adjusted mixed effects model for repeated measures (MMRM) longitudinal analyses. All postbaseline cross-sectional analyses were adjusted for age, gender, baseline score, response group, country and response group-by-country interaction. Statistical analyses were performed using sas® for Windows, version 9.1.3 (SAS Institute Inc., Cary, NC).

Outcome-related changes from baseline were analysed longitudinally up to week 8 using a MMRM approach. Within-patient errors were modelled using the unstructured covariance matrix, and the Kenward–Rodger method was used to estimate the denominator degrees of freedom for fixed effects. A type III sum-of-squares was used for the least-squares means. Longitudinal change in PGI-I was assessed using baseline CGI-S as a proxy control for baseline severity in the PGI-I MMRM model.

A MMRM sensitivity analysis using all enrolled patients was conducted for the primary outcome and a cross-sectional analysis of covariance (ANCOVA) was conducted at weeks 4 and 8 as a sensitivity analysis for all outcome measures.

Percentages and 95% confidence intervals (CIs) for patients meeting criteria for all onset, response, sustained response, remission and sustained remission measures at week 4 and 8 were reported. Maier response, HAMD_17_ overall response and HAMD_17_ sustained remission for non-responders were analysed at week 8.

The median time to onset of the indicator variables, with 95% CIs for the overall patient population, was obtained using the Kaplan–Meier method. Time to BPI-SF interference onset between IR and INR was compared using the log-rank test. Cross-sectional analyses at week 4 and 8 were conducted using an ANCOVA model to assess changes in vital signs from baseline to week 8.

## Results

### Patient disposition

**Figure 1:**
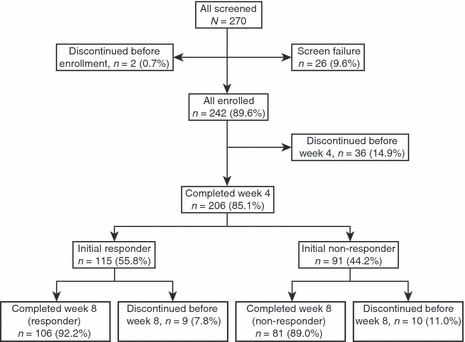
Patient disposition

Of the 242 patients enrolled in the 4-week acute therapy phase, 206 completed this initial treatment period. Of these, 115 (55.8%) patients were classified as IR and continued receiving duloxetine 60 mg/day, whereas 91 (44.2%) were classified as INR and received duloxetine 120 mg/day for an additional 4 weeks; 92.2% of the IR group (‘responders’) and 89.0% of the INR group (‘non-responders’) completed the 8-week study. Thirty-six patients discontinued the study before week 4 and did not record a primary outcome measure; these patients were ‘unclassified’. Figure 1 illustrates the patient disposition during the study. Seven IR and six INR patients were excluded from outcome analyses because of significant protocol violation or non-compliance. A further two IR and four INR patients were excluded from outcome analyses beyond week 4 caused by inadequate dose-optimisation.

### Baseline demographics

A majority of patients were women (75.2%), with a mean age of 44.9 years (Table 1). Most patients were enrolled from Canada (53.7%), with China, Korea and Brazil contributing 16.9%, 15.7% and 13.6% of patients, respectively. The most frequently prescribed previous treatments were SSRIs (177/206; 74.4%). Paroxetine and venlafaxine were the most commonly prescribed previous SSRI and SNRI treatments, respectively. Baseline characteristics were similar between IR and INR groups (Table 1).

**Table 1:** Patient baseline demographics

**Characteristic**	**IR (*n* = 115)**	**INR (*n* = 91)**	Overall*(*N* = 242)
Mean age, years (SD)	43.5 (12.9)	45.6 (12.5)	44.9 (12.5)
Female, *n* (%)	88 (76.5)	64 (70.3)	182 (75.2)
**Country**			
Brazil, *n* (%)	9 (7.8)	11 (12.1)	33 (13.6)
Korea, *n* (%)	18 (15.7)	11 (12.1)	38 (15.7)
China, *n* (%)	25 (21.7)	13 (14.3)	41 (16.9)
Canada, *n* (%)	63 (54.8)	56 (61.5)	130 (53.7)
**Previous treatment**			
SSRI, *n* (%)	83 (72.8)	69 (76.7)	177 (74.4)
SNRI, *n* (%)	31 (27.2)	21 (23.3)	61 (25.6)
**Last previous SNRI/SSRI antidepressant**			
Citalopram, *n* (%)	22 (19.3)	15 (16.7)	41 (17.2)
Escitalopram, *n* (%)	16 (14.0)	15 (16.7)	32 (13.4)
Fluoxetine, *n* (%)	3 (2.6)	4 (4.4)	9 (3.8)
Paroxetine, *n* (%)	29 (25.4)	21 (23.3)	61 (25.6)
Sertraline, *n* (%)	13 (11.4)	14 (15.6)	34 (14.3)
Venlafaxine, *n* (%)	31 (27.2)	21 (23.3)	61 (25.6)

* Overall data include patients with ‘unclassified’ response – these were the patients who discontinued before week 4 and therefore did not have a primary outcome measure (*n* = 36). IR, initial responders; INR, initial non-responders; SSRI, selective serotonin reuptake inhibitor; SNRI, serotonin norepinephrine reuptake inhibitor.

### Primary and secondary outcome analyses

**Figure 2:**
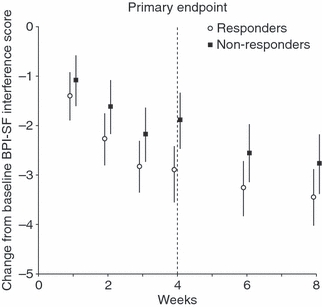
Change in BPI-SF interference score from baseline to week 8 (adjusted MMRM analysis). Overall mean difference in BPI-SF in initial responders vs. initial non-responders at week 4: 1.01 (95% CI: 0.42–1.61); p < 0.001, and responders vs. non-responders at week 8: 0.68 (95% CI: 0.03–1.33); p = 0.042. BPI-SF, Brief Pain Inventory – Modified Short Form

The mean reduction in BPI-SF interference at week 4 was greater in IRs than INRs [BPI-SF mean difference of reduction: 1.01 (95% CI, 1.61–0.42); p < 0.001]. At week 8, the difference of mean reduction from baseline BPI-SF interference between responders and non-responders was 0.68 (95% CI, 0.03–1.33; p = 0.042) (Figure 2).

**Figure 3:**
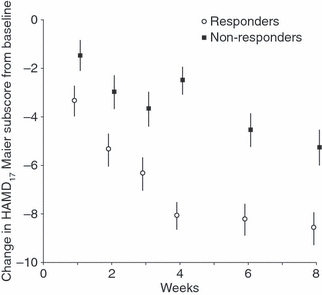
Mean change in HAMD_17_ Maier subscale score from baseline to week 8 (adjusted MMRM analysis). Mean difference in HAMD_17_ Maier score between responders and non-responders at week 8: 3.29 (95% CI: 4.10, 2.48); p < 0.001

**Figure 4:**
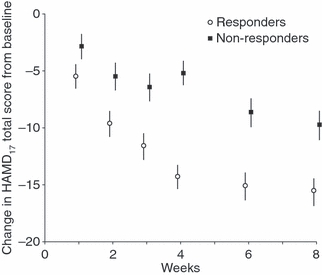
Mean change in HAMD_17_ total score from baseline to week 8 (adjusted MMRM analysis). Mean difference in HAMD_17_ total score between responders and non-responders at week 8: 5.85 (95% CI: 7.34, 4.36); p < 0.001. HAMD_17_, 17-item Hamilton Depression Rating Scale

Reductions in HAMD_17_ Maier subscale scores from baseline to week 8 were greater in responders than in non-responders (Figure 3). The mean difference in reduction between responders and non-responders at week 8 was 3.29 (95% CI, 4.10–2.48; p < 0.001) for HAMD_17_ Maier scores and 5.85 for HAMD_17_ total scores (95% CI, 7.34–4.36; p < 0.001) (Figure 4).

**Figure 5:**
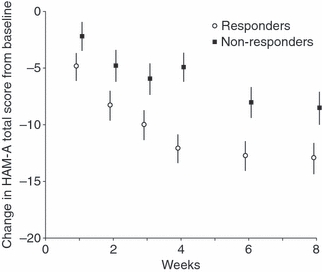
Change in HAM-A total score from baseline to week 8 (adjusted MMRM analysis). Mean difference in HAM-A total score between responders and non-responders at 8 weeks: 4.42 (95% CI: 6.04, 2.80); p < 0.001. HAM-A, Hamilton Anxiety Rating Scale

Responders also showed greater improvements in anxiety symptoms from baseline than non-responders [mean difference in reduction in HAM-A total score at week 8: 4.42 (95% CI, 6.04–2.80; p < 0.001); Figure 5]. Country and previous SSRI/SNRI were found to have no statistically significant effect on the change from baseline to week 8 in BPI-SF interference, HAMD_17_ Maier subscale score, HAMD_17_ total score or HAM-A total score.

Table 2 summarises the change from baseline to week 4 and week 8 for primary and secondary outcome measures. In general, patients were moderately ill at baseline and disease severity improved over 8 weeks, with a higher degree of improvement shown by IR and all responders (mean change in CGI-S at 4 weeks: 1.94 in IR vs. 0.57 in INR, p < 0.001; at 8 weeks: 2.52 in responders vs. 1.40 in non-responders, p < 0.001). This was also reflected by PGI-I scores over 8 weeks in all patient groups (Table 2). Improvements from baseline in SDS were shown by all patient groups (Table 2).

**Table 2:** Mean change from baseline to end-point over 4 and 8 weeks in primary and secondary outcome analyses (adjusted MRMM analysis)

	**Mean score at baseline (SD)**			**Mean reduction from baseline to week 4 (95% CI)**				**Mean reduction from baseline to week 8 (95% CI)**			
**Variable/score**	**IR (*n* = 115)**	**INR (*n* = 91)**	Overall*,†**(*N* = 242)**	**IR (*n* = 108)**	**INR (*n* = 85)**	**Mean difference between groups at week 4 (95% CI)**	**p-value**	**R (*n* = 108)**	**NR (*n* = 85)**	**Mean difference between groups at week 8 (95% CI)**	**p-value**
BPI-SF average pain	4.7 (1.9)	5.0 (2.0)	4.8 (2.0)	2.21 (1.67, 2.75)	1.03 (0.46, 1.59)	1.18 (0.60, 1.77)	0.001	2.56 (1.99, 3.13)	2.14 (1.53, 2.75)	0.42 (0.24, 1.08)	0.208
BPI-SF interference	5.2 (1.6)	5.9 (1.8)	5.5 (1.8)	2.90 (2.36, 3.44)	1.89 (1.32, 2.45)	1.01 (0.42, 1.61)	0.001	3.46 (2.89, 4.03)	2.78 (2.18, 3.38,)	0.679 (0.02, 1.33)	0.042
HAMD_17_ total	21.3 (4.5)	23.3 (4.5)	21.9 (4.6)	14.35 (13.30, 15.39)	5.20 (4.13, 6.270)	9.15 (8.04, 10.25)	< 0.001	15.64 (14.41, 16.86,)	9.79 (8.47, 11.10)	5.85 (4.36, 7.34)	< 0.001
HAMD_17_ Maier subscale	10.7 (2.6)	11.7 (2.6)	11.0 (2.7)	8.08 (7.51, 8.65)	2.54 (1.96, 3.12)	5.54 (4.96, 6.12)	< 0.001	8.58 (7.90, 9.25)	5.29 (4.56, 6.01)	3.29 (2.48, 4.10)	< 0.001
CGI-S	4.2 (0.6)	4.4 (0.6)	4.3 (0.6)	1.94 (1.74, 2.14)	0.57 (0.36, 0.78)	1.37 (1.14, 1.60)	< 0.001	2.52 (2.30, 2.75)	1.40 (1.14, 1.65)	1.13 (0.84, 1.42)	< 0.001
HAM-A total	17.9 (6.8)	21.4 (6.8)	19.2 (7.1)	12.14 (10.87, 13.41)	4.95 (3.64, 6.25)	7.19 (5.86, 8.52)	< 0.001	12.97 (11.56, 14.37)	8.55 (7.06, 10.04)	4.42 (2.80, 6.04)	< 0.001
SDS total	16.7 (7.7)	19.8 (6.8)	18.1 (7.5)	8.38 (6.50, 10.26)	2.49 (0.57, 4.42)	5.88 (4.02, 7.75)	< 0.001	9.16 (7.33, 11.00)	3.93 (2.06, 5.79)	5.24 (3.49, 6.99)	< 0.001

	**Baseline (SD)**			**Week 4 (95% CI)**				**Week 8 (95% CI)**			
	**IR (*n* = 108)**	**INR (*n* = 85)**	**IR (*n* = 108)**	**INR (*n* = 85)**			**R (*n* = 108)**	**NR (*n* = 85)**		
Mean PGI-I score‡	4.2 (0.6)	4.4 (0.6)	2.31 (2.07, 2.55)	3.20 (2.95, 3.44)	0.89 (0.64, 1.14)	< 0.001	2.12 (1.85, 2.38)	2.61 (2.33, 2.90)	0.49 (0.18, 0.81)	0.002

* Overall data include patients with ‘unclassified’ response – these were patients who discontinued before week 4 and therefore did not have a primary outcome measure (*n* = 36).

† Seven IR patients and six INR patients were excluded from outcome analyses because of significant protocol violation or non-compliance. A further two IR patients and four INR patients were excluded from outcome analyses beyond week 4 caused by inadequate dose-optimisation.

‡ Baseline CGI-S was used as a proxy measure of baseline score for PGI-I. BPI-SF, Brief Pain Inventory – Modified Short Form; HAMD_17,_ 17-item Hamilton Depression Rating Scale; CGI-S, Clinical Global Impression of Severity; HAM-A, Hamilton Anxiety Rating Scale; PGI-I, Patient Global Impression of Improvement; SDS, Sheehan Disability Scale; IR, initial responders; INR, initial non-responders; R, responders; NR, non-responders.

The majority of patients (93.3%) reported Maier onset (≥ 20% improvement) by week 8, and 68.7% and 53.0% of patients achieved HAMD_17_ response and sustained response, respectively. Over two-thirds of all patients achieved ≥ 50% improvement in BPI-SF interference and HAMD_17_ remission was achieved by 46.4% of patients at week 8 (Table 3). The BPI-SF interference onset (≥ 30% improvement) and response (≥ 50% improvement) was achieved more quickly in responders than non-responders (Log-rank test results: onset, 14 days vs. 20 days, p = 0.008; response: 21 days vs. 42 days, p < 0.001).

**Table 3:** Proportion of patients achieving secondary outcome measures at weeks 4 and 8

	**Proportion of patients achieving outcome [*n* (%**†**)]**		
**Variable (*N* = 193)**	**Week 4**	**Week 8**	**Time to event [median days (95% CI)]**
**HAMD_17_ Maier**			
Onset (≥ 20% improvement)	166 (86.0)	167 (93.3)	12 (8–14)
Response (≥ 50% improvement)	108 (56.0)	132 (73.7)	22 (21–28)
Sustained‡ response	85 (44.0)	109 (60.2)	42 (30–44)
**HAMD_17_ Total**			
Response (≥ 50% improvement)	88 (45.6)	123 (68.7)	28 (23–30)
Sustained‡ response	70 (36.3)	96 (53.0)	44 (n/a)
Remission (≤ 7)	54 (28.0)	83 (46.4)	56 (43–58)
Sustained‡ remission	42 (21.8)	63 (34.8)	41 (29–43)
**BPI-SF interference**			
Onset (≥ 30% improvement)	124 (64.2)	140 (78.2)	14 (14–16)
Response (≥ 50% improvement)	98 (50.8)	120 (67.0)	21 (21–26)

† Percentages are based on number reporting.

‡ Patients must have continued to meet the relevant criterion throughout the remainder of the study. BPI-SF, Brief Pain Inventory – Modified Short Form; CI, confidence interval; HAMD_17_, 17-item Hamilton Depression Rating Scale; n/a, not applicable.

Following duloxetine dose-optimisation to 120 mg/day, 51.3% and 44.7% of INR achieved Maier response and HAMD_17_ total response at week 8, respectively.

### Safety and tolerability

Table 4 summarises the AEs reported during the study. Overall, 153 patients (63.5%) reported TEAEs and two patients (both non-responders) experienced serious AEs during the study. One of these patients experienced worsening MDD symptoms and discontinued, the other patient experienced severe dermatitis. Eighteen patients overall [one patient (0.9%) in the responder group, two patients (2.2%) in the non-responder group and 15 ‘unclassified’ patients] discontinued the study as a result of AEs; no deaths were reported. The most common AEs reported included nausea, headache, dry mouth, dizziness, constipation, insomnia, somnolence and fatigue. There were no clinically important changes from baseline in vital signs of heart rate, blood pressure and body mass index at weeks 4 and 8 and no statistically significant differences between responders and non-responders were observed.

**Table 4:** Summary of adverse events in ≥ 5% of overall patients

**Event**	**Responders (*n* = 115)**	**Non-responders (*n* = 91)**	Overall*(*n* = 241)
Treatment-emergent AEs, *n* (%)	71 (61.7)	60 (65.9)	153 (63.5)
Discontinuations because of AEs, *n* (%)	1 (0.9)	2 (2.2)	18 (7.5)
Serious AEs, *n* (%)	0 (0.0)	2 (2.2)	2 (1.0)
**Most common AEs (≥ 5%), *n* (%)**			
Nausea	20 (17.4)	15 (16.5)	35 (17.0)
Headache	15 (13.0)	15 (16.5)	30 (14.6)
Dry mouth	12 (10.4)	14 (15.4)	26 (12.6)
Dizziness	15 (13.0)	10 (11.0)	25 (12.1)
Constipation	13 (11.3)	9 (9.9)	22 (10.7)
Insomnia	10 (8.7)	10 (11.0)	20 (9.7)
Somnolence	8 (7.0)	7 (7.7)	15 (7.3)
Fatigue	3 (2.6)	7 (7.7)	13 (5.4)

* Overall data include patients with ‘unclassified’ response – these were patients who discontinued before week 4 and therefore did not have a primary outcome measure (*n* = 36). AE, adverse event.

## Discussion

In this open-label, multicentre study, switching treatment to duloxetine 60 mg/day produced greater, clinically meaningful, improvements in interference associated with painful physical symptoms in MDD patients whose core mood symptoms improved at 4 weeks (≥ 50% improvement in HAMD_17_ Maier subscale score) compared with those whose mood did not improve. This provides an insight into the response timeline and the association between specific depressive symptoms upon switching antidepressant treatment.

Residual symptoms of MDD are often physical, including pain, and can be strong predictors of relapse (21). Patients, as in this study, may also experience residual painful symptoms following unsuccessful SSRI/SNRI treatment (2). In this ethnically and culturally diverse study, patients were included based on the presence of painful physical symptoms of at least moderate intensity (mean baseline BPI-SF average pain score: 4.8), having received treatment with SSRIs/SNRIs for at least 4 weeks for their current episode. Improvements in pain interference were observed with duloxetine at weeks 4 and 8 in most patients, with a higher degree of improvement in patients showing early significant improvements in mood. The onset of pain relief was rapid, particularly in the IR group. The median time to BPI-SF interference onset was 14 days in these patients, compared with 20 days in the INR group, suggesting a possible association between rapid pain reduction and improvements in core depressive symptoms. The present findings align with a previous assessment of time course of depression symptom improvement for duloxetine vs. placebo, where clinically meaningful symptomatic improvement was detected after 2 weeks. In that study, response was quickest on items assessing specific pain, mood, guilt, anxiety, suicidal ideation and work activities that are thought to comprise the core emotional symptoms of depression and suggested that early improvement in select symptoms may be an important indicator of long-term symptomatic resolution (22).

It has been postulated that the dual reuptake mechanism of action of duloxetine targets both the emotional and the physical symptoms of depression (1,23). The present findings suggest that the rapid and clinically significant improvement in pain shown by MDD patients switched to duloxetine is also accompanied by rapid and significant improvement in HAMD_17_ total and Maier subscore response and HAMD_17_ remission over 8 weeks, most notably in the IR group. Furthermore, significant improvements in HAM-A total score and pain interference from baseline in the responder group align with previous placebo-controlled studies demonstrating the benefits of duloxetine in treating anxiety symptoms of MDD and generalised anxiety disorder (GAD) and the PPS accompanying GAD (24,25).

Studies have shown that PPS reduces the likelihood of MDD remission (26), and a relationship between the effective treatment of pain symptoms and high HAMD_17_ remission rates was recently demonstrated (27). There is also evidence to suggest that noradrenergic or mixed reuptake inhibitor antidepressants may be more effective at relieving PPS than SSRIs (26). The presence and severity of PPS in MDD patients may therefore be an indicator of initial poor outcome following treatment with an SSRI. Addressing PPS while treating the core emotional symptoms in these patients may facilitate remission and functional improvements.

The most frequently reported TEAEs (and their prevalence) in this study were consistent with those observed in previous open-label and placebo-controlled studies of duloxetine, notably: nausea, headache, dry mouth, dizziness and constipation (28,29). Most AEs were mild and transient and 18 (7.5%) patients overall discontinued the study as a result of AEs (15 of these patients did not report a primary outcome measure). In a previous switch study of duloxetine, lower study discontinuations were reported by patients who switched treatment from a previous SSRI antidepressant compared to patients who had not been receiving treatment prior to duloxetine (30). Prior use of an SSRI/SNRI antidepressant has been suggested to act as a ‘buffer’ against AEs associated with subsequent treatment using different antidepressants of the same class (9), which may account for the lower rate of TEAE-related discontinuations with duloxetine in patients who recorded a primary outcome measure.

There are several limitations that warrant consideration when interpreting the results of this study. Firstly, this was an open-label, single-arm study with no comparator arm. Consequently, the data presented here may be limited by the biases inherent in open-label studies. In addition, the single-arm study design meant that a path analysis to identify the specific domains that contributed to the observed improvements in mood, anxiety, pain and function was not possible. Further exploration of the relationship between the individual components of depression and how these contributed to patient improvement in this study is required. Secondly, there was no duloxetine 60 mg/day treatment arm in the non-responder group during dose-optimisation; therefore, comparisons cannot be made with patients receiving duloxetine 120 mg/day who were late responders. Similarly, there was no dose-optimisation of the IR group, who may have experienced further treatment effects with a higher dose despite an early response. The lack of comparison with another antidepressant medication also limits the conclusions that can be drawn about the clinical outcomes with duloxetine following switching from previous SSRI/SNRI antidepressant treatments.

A wider degree of divergence in the secondary outcome analyses was observed between the IR and INR groups at week 4 compared with weeks 1–3 of this study. It is possible that this wider divergence was related to a more refractory patient population that improved after a further 4 weeks of dose-optimisation (late responders). This observation will be considered in a future paper, one that will also aim to understand related functional improvements and more specific attributes of response.

## Conclusion

In patients switched from SSRIs/SNRIs to duloxetine 60 mg/day for 4 weeks, initial responders on the Maier subscale showed greater improvement in pain interference than initial non-responders did. These significant responder/non-responder differences also extended to improvements in overall depressive response and remission, anxiety and functional outcome measures. Elements of core mood and pain are important residual symptoms of MDD; an early response in these symptoms after switching to duloxetine improved the chances of a clinically meaningful functional recovery.
